# Jobs Stress and Prodromal Psychosis among Employees with Different Job Occupations Abstract

**DOI:** 10.1192/j.eurpsy.2023.2323

**Published:** 2023-07-19

**Authors:** Z. Amin

**Affiliations:** Psychiatry Department (IOP), Institute of psychiatry Benazir Bhutto hospital Rawalpindi, Rawalpindi, Pakistan

## Abstract

**Introduction:**

As social stress includes social isolation, urban living, trauma and many other stressful events but social stress in context of workplace or job stress includes different factors. As in the case of social stress present at job place can be identified as a stress that is caused by planned social isolation or lack of social support at workplace. Bullying behavior of colleagues of an employee might also add up to the social stress (Øverli & Sørensen, 2016). Job stress factors also involves harassment on the hand of boss that is most often described as yelling, shouting, insulting and behaving oddly in front of other colleagues. All these factors can be quite stressful for an employee and also comes under the umbrella of social stress. This type of stress, if prolonged and adds other factors can also cause increased risk of psychoses.

**Objectives:**

Following study has certain clear objectives mentioned subsequently:

1. To investigate the prevalence of stressful work environment in association with prodromal psychosis for public and private sector employees

2. To investigate the difference between male and female employees for association of work stress with prodromal psychosis.

**Methods:**

In this Study cross sectional method will be used. The following two scales The Brief Work Stress Questionnaire to measure work stress and Prodromal Questionnaire, Brief Version (PQ-B) for measurement of prodromal psychosis will be used.

For this study a sample of 300 consisting of doctors, teachers and banking officials (100 each) will be included using convenient sampling technique. The data will be collected from different work occupations like government and private sector. Male and female sample will be collected equally.

**Results:**

The objective of the present study is therefore to inspect the symptoms of prodromal psychosis among employees belonging to different occupations. Further to explore its relationship with work stress and other social and clinical demographics. Reliability analysis was done using Cronbach’s Alpha. Internal consistency of instruments was measured by Cronbach’s Alpha. Descriptive statistics and Pearson Product Moment was also used to analyze frequencies, demographic variables percentage. Independent Sample T-test was also used for assessment of gender difference.

**Conclusions:**

This research was conducted to check the prevalence of job stress and its relation to prodromal psychosis in private and government employees in different job occupations. Other objective of this study was to explore the gender differences of job stress and prodromal psychosis among different genders as well as in different civil and private job occupations.

**Disclosure of Interest:**

None Declared

